# Feasibility of Multicomponent Training for People with Moderate to Severe Dementia Living in a Long-Term Care Home: A Social Ethical Approach

**DOI:** 10.3390/ijerph18147631

**Published:** 2021-07-18

**Authors:** Adele Kruse, Thomas Cordes, Steffen Schulz, Bettina Wollesen

**Affiliations:** Department of Human Movement Science, University of Hamburg, 20148 Hamburg, Germany; adele.kruse@uni-hamburg.de (A.K.); thomas.cordes@uni-hamburg.de (T.C.); steffen95schulz@gmail.com (S.S.)

**Keywords:** dementia, multicomponent training, long-term care home, social ethical approach

## Abstract

Multicomponent training is recommended for people with dementia living in long-term care homes. Nevertheless, evidence is limited and people with severe dementia are often excluded from trials. Hence, the aim of this study was to investigate (1) the feasibility and (2) the requirements regarding multicomponent training for people with moderate to severe dementia. The study was conducted as an uncontrolled single arm pilot study with a mixed methods approach. Fifteen nursing home residents with a mean age of 82 years (range: 75–90 years; female: 64%) with moderate to severe dementia received 16 weeks of multicomponent training. Feasibility and requirements of the training were assessed by a standardized observation protocol. Eleven participants regularly attended the intervention. The highest active participation was observed during gait exercises (64%), the lowest during strength exercises (33%). It was supportive if exercises were task-specific or related to everyday life. This study confirms that multicomponent training for the target group is (1) feasible and well accepted, and (2) to enhance active participation, individual instructions and the implementation of exercises related to everyday life is required. The effectiveness of the adapted training should be tested in future randomized controlled trials.

## 1. Introduction

In 2020, more than 50 million people worldwide were living with dementia and this number is estimated to almost double every 20 years [1]. Dementia leads to a progressive decline in function of various cognitive domains (e.g., complex attention, executive function, learning and memory, language, perceptual-motor, and social cognition) [2,3]. Furthermore, dementia is associated with an increasing need of support in activities of daily living (ADL), loss of mobility, and increased risk of falling [2,4,5]. 

However, regular physical activity (PA), defined as skeletal muscle-initiated body movement that results in energy expenditure [6], can counteract these tendencies. Regular PA has beneficial effects on a person’s health and promotes so-called healthy aging [7]. This includes a reduced loss of function, and improvements in endurance and strength in both healthy people and people with noncommunicable diseases [8]. In addition, PA also improves brain structure and function, and specific cognitive abilities [9,10]. PA, however, is significantly reduced in people with dementia when compared to age–sex matched healthy controls [4]. Consequently, it is important to promote PA in older people, regardless of their cognitive abilities. 

Furthermore, training beneficially impacts strength, mobility, and ADL in long-term care home (LTCH) residents with and without dementia [11,12,13,14,15]. Training is defined as structured (frequency, intensity, time, and type (FITT)) PA targeting the preservation or enhancement of health-related components (e.g., strength, endurance, mobility, balance) [6,16]. Especially, multicomponent training combining strength, endurance, and postural and balance exercises is recommended [12,17]. In the context of multicomponent training programs, significant improvements or positive trends in walking ability were identified in LTCH residents [18]. In addition, motor-cognitive exercises are beneficial for improving cognitive and motor performance in older adults [19,20]. Since people with dementia show increased walking insecurity, it is particularly important to develop and implement specific exercise programs in this target group [5].

However, evidence for a beneficial impact of (multicomponent) training for people with dementia is limited, as most studies are of moderate or low quality [12,14,21]. Furthermore, precise information on the combination of FITT components is unclear and seldomly reported in exercise programs for LTCH residents [22] and people with dementia [12,23]. Additionally, people with advanced cognitive impairment are often excluded from trials as they do not meet the required cognitive status [14]. Since people in the same care unit may differ largely in their cognitive status, this is a highly exclusionary approach in practice. In this context, an inclusive social ethical approach that does not exclude residents based on cognitive ability, would allow all residents of the same care unit to participate. However, this practical approach needs to be tested for feasibility and acceptance. 

To develop an effective exercise program for older adults with dementia living in a LTCH, the residents’ requirements and needs as well as the setting should be considered [21,24]. Amongst the presence of a well-known person, the specific communication, one-on-one situations, and hands-on instructions are recommended to facilitate active participation [24,25]. Interventions should be individualized to challenge the participants’ maximal capacity [15]. Moreover, individualized interventions tend to be more effective in improving the quality of life and other psychosocial components in people with dementia [26].

Although multicomponent training is recommended, the best combination of components is yet to be determined for LTCH residents with moderate to severe dementia. Hence, the critical question is: How should multicomponent training be structured to be feasible for people with moderate to severe dementia living in a LTCH? Furthermore, which are the study population’s requirements for a training intervention? Thus, the aim of the present study was to develop and evaluate the feasibility (1) and requirements regarding instruction methods, exercises, and the setting (2) of multicomponent training for people with moderate to severe dementia living in the same care unit in a LTCH. We assume that multicomponent training is feasible if the target group’s needs are met.

## 2. Materials and Methods

The study was conducted from April 2019 to June 2020 as an uncontrolled single arm pilot study to assess the feasibility of multicomponent training for LTCH residents with moderate to severe dementia. As a reporting guideline, the extended CONSORT statement for feasibility studies [27] was used. The study was approved by the local ethics committee of the faculty of psychology and human movement science of the University of Hamburg (2019_249) and prospectively registered at the German Clinical Trial Register (ID: DRKS00021438).

### 2.1. Participants

Participants were recruited at a LTCH for people with dementia in Northern Germany. This LTCH offered full stationary care for people with dementia. The costs for living and care are covered by health insurance with an additional contribution paid by the residents. Participants were living in a closed care section and family visits were allowed during the COVID-19 pandemic. It was part of the Prevention and Occupational Health in Long-Term Care Project (PROCARE) [28] and a multicomponent training group was already implemented. For this feasibility study, we included residents with dementia who, beforehand, were excluded from the pre-existing PROCARE training group because they had difficulties following verbal instructions and/or the inability to concentrate throughout the whole class. The eligibility criteria for the modified training program for people with dementia were voluntary participation, written informed consent of a legal guardian, and a diagnosis of moderate to severe dementia given by the respective physician. As this multicomponent training was designed specifically for residents who were not able to participate in the pre-existing PROCARE exercise program, no other inclusion or exclusion criteria were applied. The sample size was set to a maximum of 15 participants, which was based on the guidelines for preventive sports by the German National Association of Statutory Health Insurance Funds [29].

**Figure 1:**
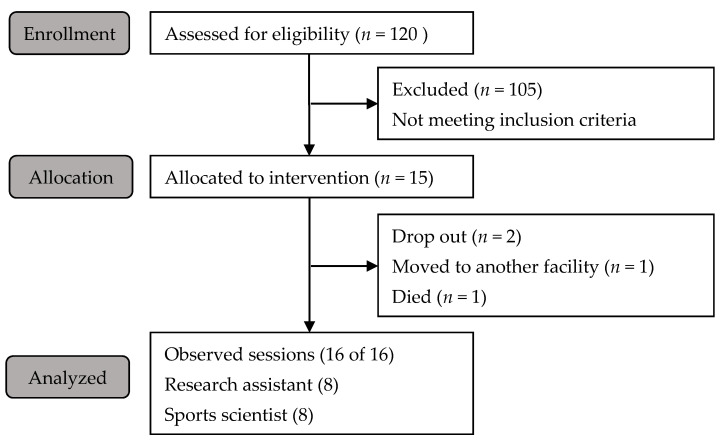
Participant flowchart.

The consulting psychologist identified N = 15 participants who met the inclusion criteria. Subsequently, the psychologist conducted an interview with the participant in the presence of the legal guardian, following the recommendations of the German Medical Association [30]. On average, participants were 82 years old (range: 75–90 years; female: 64%). A participant flowchart is presented in Figure 1. 

### 2.2. Intervention

The multicomponent training which was designed for PROCARE [28] was modified to the specific requirements of the target group (Table 1). Each session consisted of five components and was structured as follows:

The original exercise program included 32 training sessions. They were conducted over a period of 16 weeks and took place twice a week. For this study, training sessions were reduced to a total of 16 sessions because the supervising nurse had many other obligations and could only attend once a week. The sessions were conducted in a period of 16 weeks and took place once a week. Each session lasted for 45 to 60 min. 

In addition to the original program, specific exercises for people with dementia were added [31]. To enable all residents to participate, most of the exercises were performed while participants were seated. Exercise equipment included gymnastic sticks or soft balls, and everyday objects such as towels (Table A1). The program was supervised by a sports scientist. Additionally, a nurse was present during all sessions to respond to individual needs and assist with communication. The gait exercise was performed individually and accompanied by the sports scientist. Meanwhile, the nurse performed activating exercises with the nonambulatory participants (such as the coordination component). Using the observation protocol and a postsession discussion with the nurse, the research assistant and exercise scientist together continuously adjusted the exercise program. This process included optimizing the instruction methods (choice of words, position of the sports scientist, individual or group approach, and haptic/visual/auditive cues) and the adaption of exercises according to the FITT components [32]. Training sessions took place in a recreation room at the care unit. 

### 2.3. Outcome Measures

The measurements addressed the main aspects of feasibility (adherence, acceptance, exercise quantity, and performance quality). Additionally, the participants’ age and sex were provided by the facility’s psychologist. 

#### 2.3.1. Primary Outcome

Feasibility (1) of the intervention was assessed by means of a standardized observation protocol. For all sessions the retention rate (dropouts, attendance, and adverse events) and, after consultation with the nurse, the number of residents who showed signs of apathy and therefore could not actively participate, were documented. In addition, exercise quantity (the number of exercises planned and performed per session) was documented. For eight sessions a research assistant documented the performance quality divided into active (“exercise was performed correctly” or “attempts were made to perform the exercise”) and inactive (“no response”). All sessions were documented by the sports scientist and the observation of the performance quality was documented by an additional research assistant. Following each session, the sports scientist, the nurse, and the research assistant discussed the observations (Section 2.2).

Acceptability was assessed with a four-point Likert scale with smiley faces at the end of each session by the research assistant. Each item contained a statement combined with a smiley face. The scale was developed and tested in advance with a participant of a self support group by the Alzheimer’s Association Stormarn. However, as participants in the present study either did not respond to the scale or chose the first face that the research assistant explained, this survey was discontinued after four sessions.

#### 2.3.2. Secondary Outcome

Requirements regarding the instruction methods, the feasibility of exercise, and the setting (2) of the intervention were documented in an observation protocol. Half of the sessions and subsequent discussions with the nurse were documented by the sports scientist and the other half by the research assistant. Additionally, the nurse’s feedback was obtained through a questionnaire with open-ended questions after the 16-week intervention was completed.

### 2.4. Statistics

Descriptive statistics were used for quantitative measures (attendance and performance quality, exercise quantity). The activity rate (percentage of active participants in relation to all attendees) was calculated for each session and each component. In addition, the proportion of individuals with signs of apathy within the group of inactive participants was calculated. The mean (M) and standard deviation (SD) were calculated for the average number of exercises performed per session (adapted intervention of this study) and planned per session (PROCARE). Qualitative measures (field notes in blank spaces and nurse’s questionnaire) were evaluated by content analysis with category formation [33].

## 3. Results

The mean attendance rate of the 15 participants included in the study was 72% (attendance ranged between 53% and 100% per sessions). Of the eleven regular attendees, six (55%) were able to actively participate. Three (27%) regularly attending participants showed signs of severe apathy (Table 2). Two participants observed most of the time instead of taking part. Two participants sometimes left during the sessions and returned later. Reasons for participant absence were visits of relatives/friends, other appointments, or health related. There were no adverse events reported during or relating to the intervention program. 

The participants’ acceptance could not be assessed as planned. After eight sessions, the survey was stopped because the number of participants able to respond was limited. In addition, participants had difficulties identifying the smiley faces, and reading the statements and choosing one.

### 3.1. Performance Quality and Exercise Quantity

**Figure 2:**
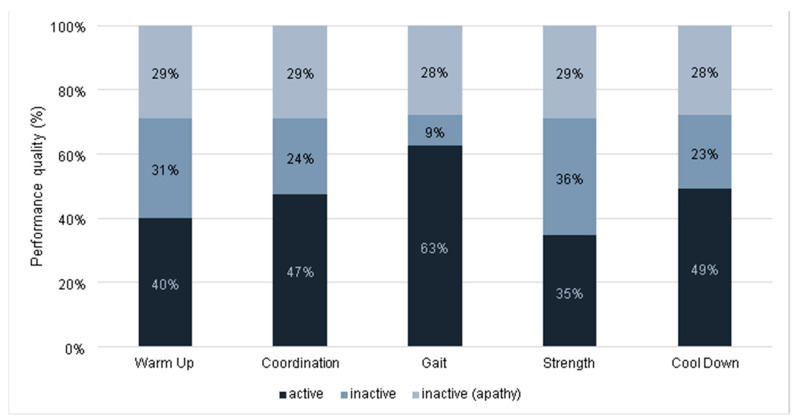
Performance quality (active/inactive/apathy) in percent for each component observed during eight training sessions.

The mean activity rate for the eight sessions assessed by the research assistant was 46% (Table A2). During these sessions, an average of 29% of participants was inactive due to apathy. For the different components, the highest activity rate was observed for the gait exercise (63%). The lowest activity rate was observed during the strength exercises (35%) (Figure 2).

On average, 23 (SD ± 5) exercises were planned for each session, of which only 19.5 (SD ± 4) exercises could be performed as the participants needed time to understand the exercises. The original PROCARE program included a mean of 35 (SD ± 12) exercises per session. In the present study, participants walked an average of 22.5 m (range 0–30 m) per session, which is 13% of the distance of the PROCARE intervention (170.6 m; range 150–240 m).

### 3.2. Secondary Outcome

The content analysis of the 16 observation protocols and the questionnaire with open-ended questions for the nurse included four main categories (“Instruction Methods”, “Exercise Design”, “Exercise Modification”, and “Setting”). 

#### 3.2.1. Instructions

It was observed that more people actively participated when the general demonstration and pictorial description of the exercise was followed by individual instructions. The attention of individual participants could be increased by addressing them by name with eye contact. When explaining the exercise individually, the sports scientist sat directly in front of the resident and demonstrated the exercise in a mirror image in combination with pictorial description and sometimes tactile cues. In addition, the nurse qualitatively reported and stated that tactile stimuli were especially important for “persons who may miss the meaning of words” to understand the exercises. The sports scientist used a rolling stool to quickly switch between participants. It was helpful to repeat the instructions several times to keep the participants’ attention.

#### 3.2.2. Exercise Design

In general, the nurse described the program as appealing and well designed. Additionally, she pointed out positively that an unknown trainer and new activities break up the routine of daily living. 

Exercises that related to everyday life (e.g., “use the towel as if you were drying your back”) or included a concrete instruction for action (e.g., “look at the neighbor on your right” instead of “turn your head to the right”) could be performed by more participants. It was supportive when the movement was performed in the field of vision. Throwing and catching, especially, activated many people. Performance was particularly high during exercises that were performed in pairs with the sports scientist (e.g., rope pull). The nurse noted that exercises were recognized by some individuals after three to four weeks due to regular repetition. Participants expressed their own ideas for exercises with reference to everyday life, music, and equipment. The equipment was generally observed as activating, but the size and design of the material also had an influence on the feasibility of exercises. All participants, including those with signs of apathy, held or engaged with cloths and small balls.

#### 3.2.3. Exercise Modification

Exercises in which body parts were moved alternately, or dual-tasks as well as grabbing and releasing material were difficult for many participants. Particularly difficult were exercises that were not related to everyday life (e.g., “turn your head to the left” or “press your hands against the outside of your thighs and try to open your thighs at the same time”). By providing a concrete action reference (e.g., “look at your left neighbor”) or tactile cues (e.g., sports scientist presses against the outer thigh), the realization of these exercises was improved. Furthermore, it was difficult for participants to perform exercises without visible movement (keeping a ball squeezed or relaxation exercises such as the body scan). It was supportive to repeatedly demonstrate the movement (repeatedly squeezing the ball) and to use active relaxation exercises (tapping massage). 

It was observed that fewer repetitions and static exercise were carried out more successfully. When switching between exercises, these had to be explained again each time. The nurse also noted that exercises became feasible through modification and after weekly repetition (three to four weeks). Additionally, she observed that individuals who are harder to reach responded with attention after seven to eight sessions. During the training, she observed more “contact looks” between participants. During the course of the training, it was observed that participants needed some time to adjust to the situation at the beginning of the training and after the gait exercise. By integrating motivational exercise equipment, the attention of the participants was refocused.

After the first session, the nurse expressed concerns about the safety of the gait exercises. Possible risk of falling and safety measures to avoid falls were discussed with the nurse, the psychologist, the sports scientist, and the research assistant. As a result, it was decided that the gait exercises be carried out with one-on-one supervision. It became apparent that it was safer to walk in an arc and not to turn around on the spot at the end of the course.

#### 3.2.4. Setting

It was observed that the armrests of the chairs were a hindrance during several exercises. Chairs without armrests would have been helpful for the residents actively participating.

It was observed that the number of active residents affected the activity level of each participant in the group. A greater number of active residents favorably influenced the activity level of habitually less active participants.

It was noted several times that one-on-one supervision would be useful. Additional staff made it possible to simultaneously activate more participants. Participants were more active when a relative assisted them throughout the training. The nurse recommended a maximum group size of eight and the presence of a second instructor to enable all participants to be active.

The aim of this study was to investigate (1) the feasibility and (2) the requirements regarding instruction methods, exercises design, and setting of multicomponent training for people with moderate to severe dementia. Overall, the multicomponent training program was accepted by most participants (mean attendance of 72%), which is similar to the adherence rate reported for training programs for people with mild to moderate dementia (mean attendance 70–80%) [12]. However, as the participants’ opinion could not be assessed, this conclusion is limited. Although the use of the faces scale was not feasible in this study, Hendriks and colleagues [34] successfully assessed the acceptance of an art intervention for people with dementia with the Visual Analog Mood Scale (VAMS, [35]). However, this finding was published subsequent to the completion of the multicomponent training [34]. The scales differ in the design and description of the faces, which is simpler in the VAMS (one explanatory word under each face). Using a similar design and a one-word description instead of complete sentences probably could have increased the feasibility of this self-report measure.

In addition to the symptoms of moderate to severe dementia, participants in this study had to cope with several health conditions, which is common in this population [36]. Thus, the training had to be adapted to the different levels of performance and attention, including walkability and apathy. 

Participant understanding of the exercises was a critical factor throughout the training and across all components. Although this effect was observed multiple times in the present study, the instructional methods are rarely discussed [12,13,15,21]. Individualized instruction combining verbal, auditory, visual, and tactile cues were most effective, which is in line with recommended communication methods in dementia care [25]. This was further facilitated in this study by the sports scientist sitting on a rolling stool and changing position frequently (Section 3.2.1). In order to ensure an individual approach from the beginning, it would have been advisable to consult the nurse in advance. However, in this study, interdisciplinary teamwork began with a discussion following the third training session. Consequently, the first three training sessions focused on familiarization. The individual instruction and the one-on-one support facilitated the engagement of more residents. This is also reflected in the training components, where the highest activity was observed during gait exercises, which were performed with one-on-one support. However, these methods are very time-consuming, which explains the low exercise quantity. In contrast to PROCARE (m = 35; SD ± 12), considerably fewer exercises could be performed (m = 20; SD ± 4). This difference is highlighted through the gait exercise, resulting in a considerably lower intensity (0–30 m per session) than in the PROCARE program (150–240 m per session; [28]). Due to less time, the intensity decreased as the proportion of ambulatory residents in the group increased. In order to integrate gait exercises and still provide an adequate intensity for both ambulatory and nonambulatory participants, tailored chair-based exercises should be integrated for those who are unable to walk [22]. To implement these simultaneous exercises, at least one trainer and one caregiver (e.g., nurse) are required. In addition, it was observed that the support of relatives during the exercises was activating. The increased activity of residents supported by their relatives during the exercises was also described by van Alphen and colleagues [24]. In the present study, external therapists and relatives were permitted to visit the LTCH during the COVID-19 pandemic. However, given the current COVID-19-related restrictions in other LTCH, relatives and external therapists were partially prohibited from entering [37]. Considering these circumstances, it would be advisable to include internal therapists and nursing staff in such an intervention. Moreover, this would create multipliers who would not be constrained by restrictions. In addition, this intervention would counteract the isolation of nursing home residents, which has been exacerbated by pandemic-related restrictions [38].

For adequate instruction according to the previously described instruction methods, the group size should be limited. With an average of eleven residents, the group was larger than in comparable studies (two to seven participants) [12]. A smaller group size of eight participants was also suggested by the nurse. 

Additionally, there is repeated emphasis in dementia research on the need to identify an appropriate configuration of the FITT components with respect to the stage of the disease [12,14]. In this study, it was found that adapting these components to the needs of people with severe dementia improved the performance quality. For example, multiset training was found to be difficult to implement because the exercises had to be explained again in each set. In contrast to multiset training with short time per exercise, single-set training provides more continuous time per exercise. With more continuous time spent on each exercise, it was possible to instruct more participants individually. Hence, the level of active participation increased and individually more exercises were performed.

In addition to identifying the best combination of FITT components [12], this study examined the exercise design. Exercises that could be well implemented related to everyday life or were task oriented. Furthermore, exercises that were initially not feasible could be modified regarding these components to increase feasibility. In this context, the high activity level during gait exercise—even though there is increasing gait insecurity [5]—could be attributed to its closeness to the daily life of ambulatory residents. In contrast, the low activity observed during strength training may reflect that these exercises were poorly related to ADL. Therefore, strength exercises in future exercise programs should be more task-oriented and related to residents’ everyday life activities, even when performed in a sitting position. 

Due to the increasing gait insecurity of elderly people and people with dementia, it was particularly important to guarantee safety during the gait exercises for this target group. Adapting the exercises to the individual’s ability and one-on-one supervision ensured this. In addition, people who needed gait aids in everyday life also used them during the gait exercises. Furthermore, assessing the risk of falls before starting the intervention, e.g., using the Tinetti balance scale, would have been advisable and should be included in future studies [39].

Due to the socioethical approach of this study, resulting in a very heterogenic group, it is essential to classify the participation rate in a differentiated way. Considering the mean active participation of 46% relative to 29% inactivity and 25% inactivity due to apathy indicates that the majority of residents without apathy participated actively. Nevertheless, a more precise scale would have been useful to quantify the heterogeneous activity levels and thereby consider the individual capacity of the residents. Consequently, engagement with equipment (holding, rope pulling with the sports scientist) and social interaction (“contact looks”) could have been included as a level of active participation as this can be considered a success for residents with signs of apathy. 

Finally, from a scientific perspective, training can be adapted and targeted more specifically to a homogeneous group. In practice, however, such an approach is highly exclusionary to many residents. Thus, the social ethical approach of this study is relevant to practice. In fact, it was observed that the activity level of the less active individuals was increased by the participation of the more active individuals in the heterogeneous group of this study. 

## 5. Limitations

Besides the small sample size, there were other limiting factors in this study. As the participants’ opinion could not be assessed by the faces scale, future studies should include a more appropiate instrument. In addition, due to the homogeneity of the group, a finer scale to assess active participation is recommended. Moreover, it would be recommendable to determine the intra- and inter-rater reliability of the two observers. In this study, however, the observation was discussed after each session. Moreover, the qualitative remarks were summarized to gain conclusions about the feasibility. Regarding the safety of the participants, it would also be advisable to assess the risk of falls before starting the intervention, for example, by using the Tinetti balance scale [39]. In the present study, this was omitted in order to ensure that participation remained a low threshold. Nevertheless, safety during walking was ensured by prior consultation with the nursing staff and additional safety measures (2:1 support, use of gait aids).

## 6. Conclusions

This study confirms that multicomponent training for people with moderate to severe dementia is feasible. Since a highly individualized approach seems to increase the level of activity, the group size should be kept to a minimum of eight participants. For adequate supervision of the training, an appropriately qualified instructor (e.g., sports scientist) assisted by a caretaker known to the participants is needed. To enable as many LTCH residents as possible to actively participate, exercises should be task-oriented, related to everyday activities and tailored to the individual residents’ capacities. Further research should implement the identified requirements of this study to test the effectiveness of this optimized multicomponent training in future randomized controlled trials.

## Figures and Tables

**Table 1:** Comparison of the multicomponent training PROCARE and PROCARE dementia.

Component	PROCARE Original	PROCARE Dementia
Duration	32 sessions in 16 weeks	16 sessions in 16 weeks
Warm-up	5–10 min standing	5 min seated
Balance, coordination, and cognition	10 min	10–15 min
Gait exercise	20 min	5 min per person If not able to walk: chair-based exercise with the nurse
Strength	10 min	10 min
Cool down	5–10 min	5 min

**Table 2:** Baseline characteristics of participants.

	Participants	Female	Male
Included	15	10	5
Ambulatory	10	5	5
Signs of apathy	3	3	0
Regularly active	6	3	3

## Data Availability

The data presented in this study are available on request from the corresponding author (B.W.).

## Appendix A

**Table A1:** Exercise examples of a training session with progression of exercises.

Training Components	Level 1	Level 2	Level 3
Mobilization and warm-up	Moderate mobility and range of motion exercises for the wrists, hip, shoulders, knees, and ankles	Moderate mobility and range of motion exercises for the wrists, hip, shoulders, knees, and ankles	Moderate mobility and range of motion exercises for the wrists, hip, shoulders, knees, and ankles
Coordination and motor-cognitive exercises	Waving, swinging back and forth, circling in the air with a cloth	Throw up a cloth and catch it again	Pass cloths to the trainer and catch them again
	Cognitive-motor tales (incorporated movement into stories, e.g., a day at the long-term care home)	Cognitive-motor tales (incorporated movement into stories, e.g., a visit to the garden)	Cognitive-motor tales (incorporated movement into stories, e.g., a trip to the beach)
Gait exercise	15 m walk, pleasant pace, there and back	15 m walk, pleasant pace, there and back	15 m walk, pleasant pace, there and back with instruction “stop and go”
		15 m walk at a brisk pace	15 m walk at a brisk pace with instruction “stop and go”
Strength and aerobic exercises	Bending and stretching knees, 20 repetitions	Bending and stretching knees, 2 × 20 repetitions	Bending and stretching knees, 3 × 15 repetitions
	Rowing with stick, 15 repetitions	Rowing with stick, 2 × 15 repetitions	Rowing with stick, 3 × 15 repetitions
	Rotation of upper body, 10 repetitions	Rotation of upper body, 2 × 15 repetitions	Rotation of upper body, 3 × 15 repetitions
	Strength exercises with the towel (e.g., compress between knees) 10 × 3 s.	Strength exercises with the towel, 10 × 5 s.	Strength exercises with the towel, 15 × 5 s.
	Biceps curls and making a fist, 1 × 15 repetitions	Biceps curls and making a fist, 2 × 15 repetitions	Biceps curls 1–2 kg weights, 2 × 15 repetitions
Cool down	Stretching exercises and progressive muscle relaxation	Stretching exercises and progressive muscle relaxation	Stretching exercises and progressive muscle relaxation

**Table A2:** Mean activity rate (%) for observed sessions divided into four categories.

	Active	Partially Active	Inactive	Inactive (Apathy)
Activity (total)	31%	15%	25%	29%
Warm up	24%	17%	31%	29%
Coordination	28%	20%	24%	29%
Gait Exercise	54%	9%	9%	28%
Strength Exercise	27%	8%	36%	29%
Cool Down	23%	26%	23%	28%
